# Comparative Analysis of the Transcriptome in Tissues Secreting Purple and White Nacre in the Pearl Mussel *Hyriopsis cumingii*

**DOI:** 10.1371/journal.pone.0053617

**Published:** 2013-01-14

**Authors:** Zhiyi Bai, Hanfeng Zheng, Jingyun Lin, Guiling Wang, Jiale Li

**Affiliations:** 1 Key laboratory of Freshwater Aquatic Genetic Resources, Shanghai Ocean University, Ministry of Agriculture, Shanghai, China; 2 East China Sea Fisheries Research Institute, Chinese Academy of Fishery Sciences, Shanghai, China; 3 Aquaculture Division, E-Institute of Shanghai Universities, Shanghai Ocean University, Shanghai, China; Temasek Life Sciences Laboratory, Singapore

## Abstract

The triangle sail mussel *Hyriopsis cumingii* (*Lea*) is the most important mussel species used for commercial freshwater pearl production in China. Mussel color is an important indicator of pearl quality. To identify genes involved in the nacre coloring, we conducted RNA-seq and obtained 541,268 sequences (298 bp average size) and 440,034 sequences (293 bp average size) in secreting purple and white nacre libraries (P- and W-libraries), respectively. The 981,302 Expressed Sequence Tags (ESTs) were assembled into 47,812 contigs and 289,386 singletons. In BLASTP searches of the deduced protein, 22,495 were proteins with functional annotations. Thirty-three genes involved in pearl or shell formation were identified. Digital expression analysis identified a total of 358 differentially expressed genes, and 137 genes in the P-library and 221 genes in the W-library showed significantly higher expression. Furthermore, a set of SSR motifs and SNPs between the two samples was identified from the ESTs, which provided the markers for genetic linkage, QTL analysis and future breeding. These EST sequences provided valuable information to further understand the molecular mechanisms involved in the formation, color determination and evolution of the pearl or shell.

## Introduction

Pearl and nacre, or mother of pearl, have long been appreciated for their beauty, but have only been scientifically studied for the past 150 years [1]. As a result of this research, nacre is not just of interest for its aesthetic qualities, but also as a material of exceptional performance when compared to the properties of its component parts [2]. For those engaged in fabricating biomimetic products, it has become a challenge to replicate the structure of the iconic biomineral [3]. Recent ultrastructural advances have shown that each nacre tablet has a hierarchical organization [4] and is composed of nanodomains [5]. In parallel, biochemical characterizations have indicated that the nacre matrix is composed of a large set of macromolecular components, such as chitin, hydrophobic “framework” proteins, proteins with elastomeric properties, soluble proteins and glycoproteins [6]. For several years, new dynamic models of nacre formation have been used to conciliate both biochemical and ultrastructural data. Particular emphasis has been placed on the prominent role played by chitin in spatially structuring the organic framework, the distribution of key biochemical functions at the surfaces of nacre tablets, the existence of a transient precursor amorphous phase, and the growth of nacre tablets in a hydrophobic gel, which hardens and becomes insoluble when nacre tablets coalesce [7]. However, the molluscan shell forming secretome is rapidly evolving. This is particularly pronounced in the genes encoding secreted proteins, many of which are likely to contribute directly to shell formation [8]. By directly comparing the transcriptomes of nacre forming cells in a bivalve and a gastropod, dramatic differences in the gene sets used to biofabricate the nacreous layer of the shell were identified [9].

For pearl oysters or mussels, previous research has focused primarily on marine species, especially *Pinctada* species [10], [11]. The triangle sail mussel *Hyriopsis cumingii* (*Lea*), a freshwater pearl mussel in China, has a different evolutionary position than that of the seawater pearl oyster [12]. Other than the calcite prism of seawater oysters, freshwater mussel prisms are aragonite. Today, China is the largest commercial producer of freshwater pearls, producing 1,500 tons in 2005. So far, the triangle sail mussel is the most important and widely used pearl mussel, followed by a hybrid between the sail mussel and *Hyriopsis schlegelii* introduced from Japan [13]. Pearl quality is correlated with five variations, including size, shape, color, luster and surface complexion, of which color is a subjective indicator. Purple pearls hold the greatest value of any freshwater pearls, with similar quality characteristics [14]. Recently, increasing attention has been paid to research on pearl coloring, primarily focused on metal ion coloring, organic pigment coloring and structural coloring [15], [16].

Expressed Sequenced Tags (ESTs) present a valuable resource for research and breeding, as they provide comprehensive information regarding the dynamics of the freshwater pearl mussel transcriptome. ESTs have played significant roles in accelerating gene discovery, including gene family expansion [17], improving genome annotation [18], elucidating phylogenetic relationships [19], facilitating breeding programs for aquaculture animals by providing SSR and SNP markers [20], [21], and large-scale expression analysis [22]. In addition, ESTs are a robust method for rapid identification of transcripts involved in specific biological processes. Due to cost limitations, we have sequenced and analyzed only 5,019 ESTs from a mantle cDNA library using first generation sequencing technology [23]. So far, no other systematic studies on the entire transcriptome in freshwater pearl mussels have been performed, and we are lagging in our understanding of the molecular mechanisms underlying pearl or shell formation and color determination in freshwater mussels.

In our laboratory, the purple line mussels (P-line, mussels with purple nacre inside the shell) and white line mussels (W-line, mussels with white nacre inside the shell) have been under mass selection since 2001, and reached the fourth selected generation in 2009. The availability of inbred mussels with different nacre color permitted us to use comparative genetics to unveil the molecular mechanisms of nacre coloring and nacre formation. Recent advances in next generation sequencing technologies allowed us to generate large scale ESTs efficiently and cost-effectively. In this study, we report the generation of more than 981,302 high quality freshwater pearl mussel ESTs from tissues secreting purple and white nacre, using Roche-454 massive parallel pyrosequencing technology. These ESTs were clustered and assembled into 222,158 unisequences with open reading frames, which were further annotated in this study. Thirty-three genes potentially implicated in the biomineralization process were identified. We then performed a comparative digital expression profiling analysis to systematically characterize differences in mRNA expression levels between the tissues secreting purple and white nacre, in an attempt to identify genes involved in nacre color determination. Furthermore, putative SNP and SSR markers were identified from these ESTs.

## Materials and Methods

### Ethics Statement

The handling of mussels was conducted in accordance with the guidelines on the care and use of animals for scientific purposes set by the Institutional Animal Care and Use Committee (IACUC) of Shanghai Ocean University, Shanghai, China.

### Mussel tissues samples

**Figure 1:**
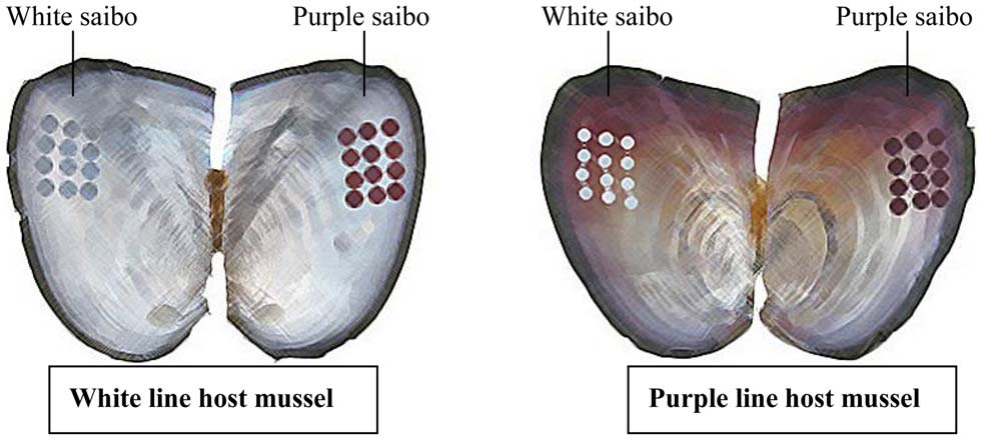
Grafting diagram of host mussels and donor mussels.

Live freshwater pearl mussel individuals were collected from Weiwang Pearl Farm of Jinhua, Zhejiang Province, China. The P-line and W-line mussels have been under mass selection since 2001 and reached the fourth selected generation in 2009. Young mussels were reproduced in May and reached a shell length of approximately 10 cm before they were grafted in November 2009. Twelve purple saibos (from the P-line mantle) and twelve white saibos (from the W-line mantle) were grafted into the left and right mantles of each P- or W-line host mussel, respectively (Figure 1). After grafting, host mussels were cultured in the same pond until sampling in October 2011. Healthy mussels of the same size were chosen and used for the collection of mantle (section for secreting nacre) from the P- and W-line host mussels, pearl sac secreting purple pearl in P- and W-line host mussels and pearl sac secreting white pearl in P- and W-line host mussels. All samples were stored in liquid nitrogen for the following experiments.

### RNA extraction, cDNA synthesis and sequencing

Each frozen sample was ground in a mortar with liquid nitrogen, and total RNA was isolated using TRIzol reagent (Invitrogen, USA) according to the manufacturer's protocol. The final total RNA was dissolved in 200 µL RNase-free water. The total RNA concentration was determined using a NanoDrop (Thermo Scientific, USA), and the RNA integrity value (RIN) was checked using the RNA 6000 Pico LabChip of an Agilent 2100 Bioanalyzer (Agilent, USA). In the P-library, total RNA extracted from the P-line mussel mantle, pearl sac secreting purple pearl in P-line host mussel and pearl sac secreting purple pearl in W-line host mussel were pooled together in the ratio 2∶1∶1. In the W-library, total RNA extracted from the W-line mussel mantle, pearl sac secreting white pearl in P-line host mussel and pearl sac secreting white pearl in W-line host mussel were also pooled together in the ratio 2∶1∶1. Total RNA from both libraries was incubated with 10 U DNase I (Ambion) at 37°C for 1 hr, and nuclease-free water was added to bring the sample volume to 250 µL. The mRNA was further purified with a MicroPoly(A) Purist Kit (Ambion, USA) according to the provided protocol. The mRNA was dissolved in 100 µL of RNA Storage Solution. The final concentration of RNA was determined using a NanoDrop.

Double-stranded cDNA was synthesized from mRNA according to Ng's full-length cDNA synthesis protocol with some modifications [24]. A GsuI-oligo dT primer was used to prime the first-strand cDNA synthesis from 10 µg of mRNA, using 1000 units of Superscript II reverse transcriptase (Invitrogen, USA). After incubation at 42°C for 1 hr, the 5′-CAP structure of mRNA was oxidized by NaIO4 (Sigma, USA) and ligated to biotin hydrazide, which was used to select complete mRNA/cDNA by binding Dynal M280 beads (Invitrogen, USA). After the second strand cDNA synthesis, the polyA and 5′ adaptor were deleted by GsuI digestion. cDNA size fractionation was performed using a cDNA size fractionation column (Agencourt, Germany). Each cDNA fraction larger than 800 bp was sonicated to a range of 300–800 bp.

The prepared cDNAs were transformed into single-stranded template DNA (sstDNA) libraries by using the GS DNA Library Preparation kit (Roche Applied Science, Switzerland). sstDNA libraries were clonally amplified in a bead-immobilized form using the GS emPCR kit (Roche Applied Science, Switzerland). A half-plate sequencing run was performed for each library on the 454 Genome Sequencer FLX instrument.

### EST Assembly and bioinformatics analysis

The 454 sequencing reads were filtered to remove low quality reads using a program developed in-house. The qualified reads from different tissues were pooled together and assembled by CAP3 using default parameters [25]. Reads from different tissues could be distinguished from read name in assembled “Ace” file. Open reading frames were identified using a program developed in-house based on ‘GetORF’ from EMBOSS [26]. Finally, open reading frames were identified from 46,871 contigs and 175,287 singlets. Gene annotation was performed using a BLASTP search against Swiss-Prot and GenBank (NCBI) non redundant protein databases with an E-value of 1e^−3^, and the best annotation was selected for the result. Gene ontology analysis was performed using GoPipe [27] through BLASTP against the Swiss-Prot and TrEMBL databases with an E value of 1e^−3^. This analysis found 9,135 deduced protein sequences that matched 61,637 GO terms. BLASTP was also used to align deduced protein sequences to the Kyoto Encyclopedia of Genes and Genomes (KEGG) [28] and Clusters of Orthologous Groups (COG) (http://www.ncbi.nlm.nih.gov/COG/) with an E-value of 1e^−3^ [29] to predict possible functional classifications and molecular pathways.

We used XSTREAM [30] to isolate genes coding for proteins with tandem-arranged repeat units. In our search, deduced protein sequences with a coding region shorter than 150 bp were discarded. The settings used in this analysis were used as the default settings, i.e., degeneracy = 0, TR significance = high, and min consensus match = 0.8. Deduced proteins containing tandem-arranged repeat units with enriched Asp, Gly and Lys residues were picked out manually.

### The identification of differentially expressed genes, SNPs and SSRs

Reads belonging to different tissues were counted based on “Ace” file of CAP3 assembly result. Then the reads number of each tissue in one contig was transformed into RPKM (Reads Per Kilo bases per Million) based on the total reads number of each stage [31], and differentially expressed contigs between the two tissues were identified by a DEGseq package using the MARS (MA-plot-based method with Random Sampling model) method [32]. FDR≤0.001 and the absolute value of log_2_Ratio≥1 were used as the threshold to judge the significance of the contig expression difference.

SNPs in the cDNA sequences between the two samples were identified with PolyBayes [33]. To eliminate errors introduced by PCR amplification during the cDNA synthesis step and homopolymer errors introduced by the 454 pyrosequencing technology, and to distinguish true SNPs from allele differences, we further filtered the PolyBayes results and only kept SNPs meeting the following criteria: 1) at least 2×coverage at the potential SNP site for each sample, 2) at least 60% of sequences were the same base at the potential SNP site for each sample, 3) not an indel site surrounded by a long stretch (> = 3) of homopolymers.

The set of the unisequences was screened for microsatellites using the software MISA (MIcroSAtellite, http://pgrc.ipk-gatersleben.de/misa/). For this study, the criteria for SSRs were set as sequences having at least ten repeats of di-nucleotide, six repeats of tri-nucleotide and five repeats for all other repeats (tetra-, penta-, and hexa-nucleotide) [34].

## Results and Discussion

### EST sequencing and general characteristics

We conducted RNA-seq for the P- and W- cDNA libraries, resulting in 541,268 sequences with an average size of 298 bp and 440,034 sequences with an average size of 293 bp (Table 1), respectively. Sequences in the P-library ranged from 18 to 735 bp, with 22.6% of the sequences longer than 400 bp. Sequences in the W-library ranged from 18 to 809 bp, with 46.3% of the sequences longer than 400 bp. Small open reading frames (smORFs) might encode translated and biologically active peptides [35], so we included the short sequence in this study. The 981,302 ESTs were assembled into clusters using CAP3, which produced 47,812 contigs and 289,386 singletons. Data were archived at the NCBI Sequence Read Archive (SRA) under accession SRA056515. The average contig length was 634 bp and ranged from 42–10,137 bp, while the average total read was 14.5. The 46,871 contigs and 175,287 singlets were found and open reading frames (ORFs) were extracted using a program developed in-house based on ‘GetORF’ from EMBOSS. All of the ORF sequences were translated into protein sequence.

**Table 1:** Summary of sequencing results.

Feature	Number
Read number	981,302
-P-library	541,268
-W-library	440,034
Number of assembled reads	691,916
- P-library	340,491
- W-library	351425
Average read length (bp)	296
- P-library	298
- W-library	293
Number of contigs	47,812
Average length of contigs(bp)	634
-Max.	10,137
-Min.	42
Average reads of contigs(bp)	14.5
-Max.	16,495
-Min.	2

### Functional annotation of the transcriptome

**Figure 2:**
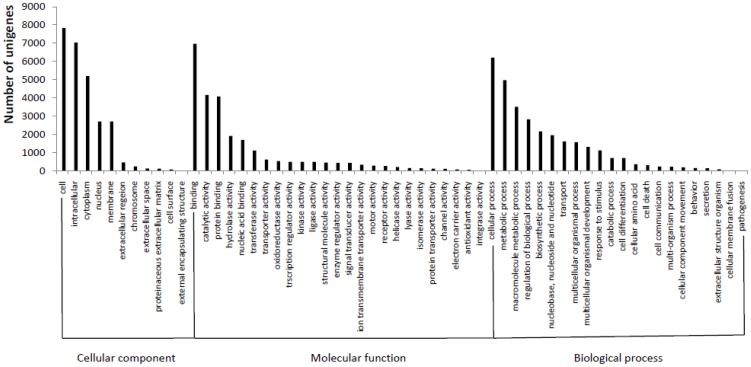
Gene Ontology classification of deduced protein sequences.

BLASTP searches of the deduced protein sequences in the Swiss-Prot and Genbank non redundant protein databases revealed 22,495 protein sequences with a functional annotation. Gene Ontology (GO) terms were further assigned to virtually deduced protein sequences based on their sequence similarities to known proteins in the Swiss-Prot and TrEMBL databases. A total of 9,135 virtually deduced protein sequences (40.6%) were assigned 61,637 GO terms: 26,550 in the biological processes category, 25,730 in the molecular function category and 30,442 in the cellular component category. Of the deduced protein sequences categorized as cellular components, 29.5% were categorized as “cell,” 26.5% as “intracellular,” and 19.6% as “cytoplasm.” The majority (27.1%) of the unisequences categorized as molecular functions were involved in binding, followed by those involved in catalytic activity (16.2%) and protein binding (15.9%). The most represented biological processes were cellular processes and metabolic processes, which comprised 20.4% and 16.3%, respectively. An overview of their classification is shown in Fig. 2.

Using KEGG, 5,023 deduced protein sequences were assigned into specific pathways (Fig. S1). Of these deduced protein sequences, 24.7% were included in metabolism processes, and there were three major subgroups involved in carbohydrate metabolism, lipid metabolism and amino acid metabolism. The other deduced protein sequences were assigned into pathways involving genetic information processing (15.5%), cellular processes (11.5%), organismal systems (17.5%), human diseases (16.7%), and environmental information processing (7.9%).

**Figure 3:**
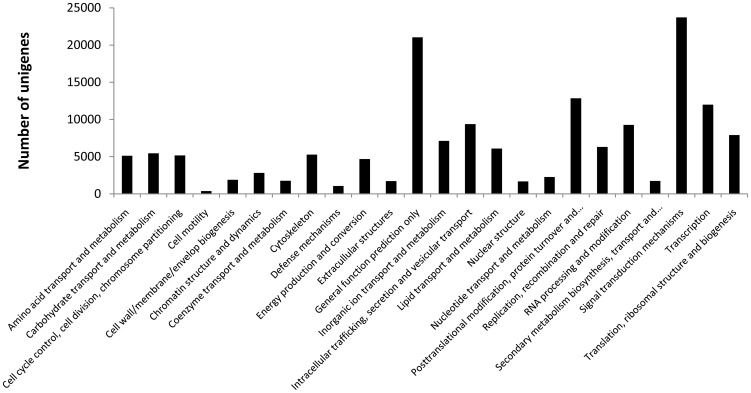
COG classification of deduced protein sequences.

Domains can be thought of as distinct functional and/or structural units of a protein. These two classifications coincide rather often, and an independently folding unit of a polypeptide chain also carries a specific function. Domains are often identified as recurring sequence or structural units, which may exist in various contexts. In molecular evolution, such domains may have been utilized as building blocks, and may have been recombined in different arrangements to modulate protein function. CDD defines conserved domains as recurring units in molecular evolution, which can be determined by sequence and structure analysis [29]. All deduced protein sequences were annotated for conserved domains using COG searches against the clusters of orthologous groups for the eukaryotic complete genomes database with an E-value of >1e^−3^. In result, 23,727, 12,851, 11,986 protein sequences were respectively assigned COG items in signal transduction mechanisms, posttranslational modification, protein turnover, chaperones and transcription. An overview of the classification is shown in Fig. 3.

### The identification of transcripts encoding proteins involved in the biomineralization process

Nacreous and prismatic are the two most common molluscan shell textures. Nacre is still one of the most studied shells for several reasons, including its economic value and mechanical properties. As the currently accepted “chitin-silk fibroin gel-acidic macromolecule” model, nacre is a biocomposite involving three components: minerals, polysaccharides (chitin), and proteins (glycoproteins, silk fibroin, and others) [7]. Here, we identified and classified the genes involved in the biomineralization process, including six genes related to chitin biosynthesis and/or modification, five moderately acidic shell protein genes, two basic shell protein genes, ten genes involved in calcium metabolism, six genes involved in metal metabolism, two matrix protein genes and two genes binding to or cross-linking the matrix.

Nacre assembly begins at the molecular level with the fabrication of the polysaccharide chitin (N-acetyl-2-glucosamine), which is secreted by the animal from its mantle into the liquid filled extrapallial space between the mantle and the shell. The latter is sandwiched by layers of silk fibroin-like proteins, which are enriched in Gly alone or in Gly and Ala [36]. No silk fibroin-like protein was identified in this study, but we searched the deduced proteins contained in the tandem-arranged repeat units with enriched Gly residues (see section “Proteins with tandem-arranged repeat units”). As the main components of organic interlamellar sheets in nacre, highly ordered and aligned beta-chitin constructs the scaffold as the template for nucleation of the calcium carbonate aragonite crystals [37]. In this study, six genes related to chitin biosynthesis and/or modification were found: chitin synthase, chitin binding peritrophin-A, chitin deacetylase 5, chitin deacetylase 1, chitinase and chit3 protein (Table 2).

**Table 2:** Genes involved in the biomineralization process.

Putative function	Gene name (Query)	Accession No.	E-value	Species
**Chitin biosynthesis and modification**	Chitin synthase	Contig12395_52	0.0	*Pinctada fucata*
	Chitin binding peritrophin-A	Contig10542_8	2e-06	*Ixodes scapularis*
	Chitin deacetylase 5	Contig25082_26	7e-60	*Papilio polytes*
	Chitin deacetylase 1 precursor	Contig28908_5	1e-18	*Cherax quadricarinatus*
	Chitinase	Contig19449_4	3e-63	*Crassostrea gigas*
	Chit3 protein	p-G9PKTWR01BW1TH_9	2e-39	*Crassostrea gigas*
**Molluscan Shell Proteins**	Pif 177	Contig14390_10	3e-18	*Pinctada fucata*
	Nacrein B3	Contig43912	5e-09	*Pinctada margaritifera*
	Nacrein B4	Contig23367	6e-10	*Pinctada margaritifera*
	Tyrosinase	Contig46530_5	5e-13	*Illex argentinus*
	Dermatopontin 2	Contig40684_4	5e-45	*Haliotis diversicolor supertexta*
	Perlucin 6	p-G9NRHGJ02H3KTQ_2	3e-13	*Haliotis diversicolor*
	PfN23	Contig24025_3	3e-21	*Pinctada fucata*
**Metal metabolism**	Calreticulin	Contig20109_3	0	*Pinctada fucata*
	Calmodulin	Contig41460_17	3e-15	*Haliotis discus discus*
	Calcium-ATPase	w-G90OYPM02IM8L9_3	4e-10	*Loligo forbesii*
	Calcitonin receptor	Contig255_4	7e-30	*Crassostrea virginica*
	Voltage-dependent L-type calcium channel alpha-1 subunit	w-G9CKNNJ01BTOZ8_3	1e-57	*Lymnaea stagnalis*
	Calcium homeostasis endoplasmic reticulum protein	Contig15522_24	1e-78	*Danio rerio*
	Histidine rich calcium binding protein	Contig24603_3	9e-18	*Mus musculus*
	Calcium-binding atopy-related autoantigen 1	Contig28072_6	7e-42	*Acromyrmex echinatior*
	Calcium and integrin-binding protein 1	p-G9PKTWR01B78VK_1	8e-17	*Harpegnathos saltator*
	Calcium binding protein	Contig44288_9	2e-06	*Euglena gracilis*
	Multicopper oxidase	contig4154_8	2e-152	*Crassostrea gigas*
	Mg2+ and Co2+ transporter	Contig3824_6	1e-24	*Ixodes scapularis*
	Ferritin	p-G9NRHGJ02F62Y3_6	0	*Hyriopsis cumingii*
	Cadmium metallothionein	Contig3396_4	9e-28	*Schistosoma mansoni*
	Matrix metalloproteinase	Contig5483_17	6e-43	*Crassostrea gigas*
	Astacin-like protein	p-G9NRHGJ02F543V_9	2e-05	*Pinctada fucata*
**Others**	Dentin matrix protein	p-G9PKTWR01A8WP0_9	3e-27	*Ixodes scapularis*
	Fras1 related extracellular matrix protein	Contig20564_10	1e-33	*Schistosoma mansoni*
	Perlecan	Contig21930_8	3e-90	*Homo sapiens*
	Laminin	p-G9NRHGJ02IXPYS_3	1e-59	*Bombus impatiens*
	Carbonic anhydrase	Contig25806_12	6e-29	*Loligo bleekeri*

Shell proteins not only participate in the construction of the organic nacre framework, but also control the nucleation and growth of aragonitic crystals, as well as the polymorph specificity of calcium carbonate in nacre. Currently, clearly identified shell matrix protein families are not available, so we chose to present the shell proteins in groups according to their theoretical isoelectric point [6]. In calcitic prisms, the shell matrix is particularly acidic [38], so it seems reasonable that extremely acidic shell proteins were not isolated from tissues secreting nacre in this study. Five moderately acidic shell proteins, including Pif-177, nacrein B3, nacrein B4, tyrosinase and dermatopontin 2, were found in this study (Table 1). Pif-177 was identified in the nacre shell of *P. fucata* and specifically binds to aragonite crystals. Results from immunolocalization, RNA interference and in vitro calcium carbonate crystallization strongly indicate that Pif-177 regulates nacre formation, making Pif-177 the first mineralization protein in this species whose function was identified in vivo [39]. Nacrein is the first protein, which was proved to work as an enzyme. Nacrein exhibits several GXN repeats (where X is frequently D, N, or E), flanked by two carbonic anhydrase-like subdomains. In situ hybridization studies show that nacrein is ubiquitous and is likely to display the same functions in the prismatic and nacreous layers [40], [41]. Tyrosinase, a particular phenoloxydase, is a copper containing enzyme that binds oxygen. It is involved in the oxidation of phenol groups of tyrosine residues, which results in the formation of melanin. One tyrosinase found in *Pinctada fucata* seems to be involved in the pigmentation of the prismatic layer and may be included in the prismatic layer [42]. The dermatopontin gene is ancient because of its widespread repartitioning in several metazoan lineages and its general function in extracellular matrix assembly. It has been suggested that the snail dermatopontin has a role in spatially organizing the shell matrix [43]. Contrary to the acidic proteins, proteins with a basic pI were rather unexpected components of the molluscan shell matrix. However, two basic shell proteins, perlucin 6 and PfN23, were found in this study. Perlucin 6 is able to accelerate the nucleation of CaCO3 layers on top of calcite surfaces, and is incorporated as an intracrystalline component of the neosynthesized crystals [44]. PfN23 is a key accelerator in the control of crystal growth in nacre [45].

Calcium carbonate accounts for more than 95% of the nacre weight. Therefore, calcium metabolism plays an important role in pearl formation. Considering the importance of calcium homeostasis in eukaryotic cells, the regulation of calcium content in the cell is crucial. In this study, ten genes involved in calcium metabolism were identified: calreticulin, calmodulin, calcium-ATPase, calcitonin receptor, voltage-dependent L-type calcium channel alpha-1 subunit, calcium homeostasis endoplasmic reticulum protein, histidine rich calcium binding protein, calcium-binding atopy-related autoantigen 1, calcium and integrin-binding protein 1 and calcium binding protein. Carbonic anhydrase (CA) catalyzes the reversible hydration of carbon dioxide and has been found to be associated with calcification in a number of invertebrates. One carbonic anhydrase was found in this study. In addition to calcium, many other metals are contained in nacre. In a previous study [46], higher concentrations of Zn, Mg, Ti, V, Ag and Co were found in purple pearls as compared to white pearls. In this study, six genes involved in metal metabolism were also identified: multicopper oxidase, Mg^2+^ and Co^2+^ transporter, ferritin, cadmium matallothionein, matrix metalloproteinase and astacin-like protein.

The extracellular matrix is an integrated system regulating protein–mineral, protein–protein and feedback interactions between biominerals and the calcifying epithelium that synthesizes them [47]. In this study, two matrix proteins, dentin matrix protein and Fras1 related extracellular matrix protein, were identified. Additionally, two proteins that bind to or cross-link the matrix, perlecan and laminin, were identified.

### Proteins with tandem-arranged repeat units

**Figure 4:**
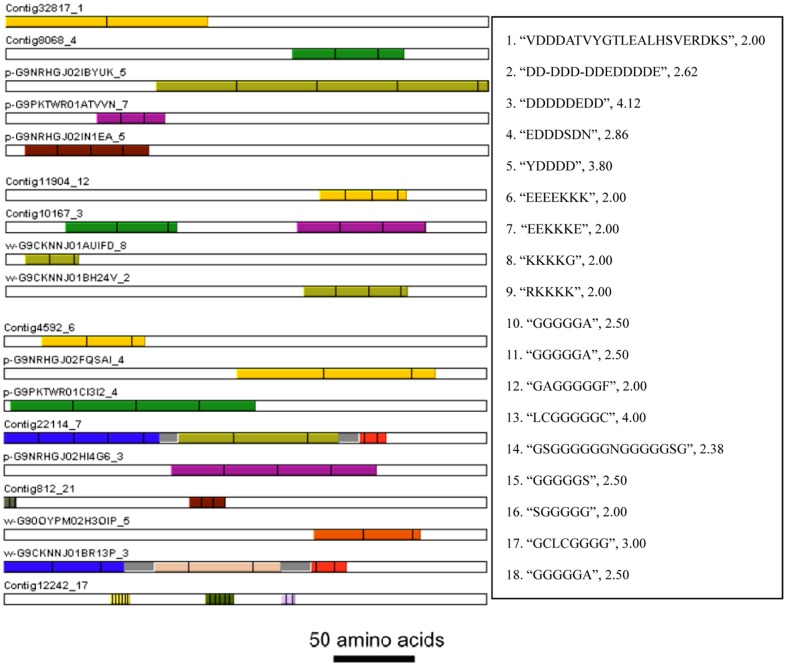
Schematic of deduced proteins contained tandem-arranged repeat units (1–5) enriched Asp residues, (6–9) enriched Lys residues, (10–18) enriched Gly residues. Each motif and copy number was shown on the right from up to down. Bars = 50 amino acids.

An important feature of biomineralization-related proteins in pearl bivalves is their modularity, which means that they are organized in different functional domains. Many of the domains are composed of tandem-arranged repeat units. XSTREAM analysis found that 2,502 (1.1%) of deduced proteins contained tandem-arranged repeat units. In this study, nine deduced proteins contained tandem-arranged repeat units with no less than four uninterrupted Lys residues. In addition, forty-eight deduced proteins contained tandem-arranged repeat units with no less than two uninterrupted Asp residues and forty-six deduced proteins contained tandem-arranged repeat units with no less than two uninterrupted Lys residues. Further, five deduced proteins contained tandem-arranged repeat units with no less than three uninterrupted Asp residues, and four deduced proteins contained tandem-arranged repeat units with no less than three uninterrupted Lys residues (Fig. 4).

The latest achievements in this field suggest a multistep strategy of phase transformation and highlight the enrollments of transient amorphous precursors in biomineralization [48]. It is believed that these amorphous phases are the reservoirs for crystallization and the mechanical strengtheners during biomineral formation. However, the pure amorphous calcium minerals are highly unstable in humid environments and theoretically, they could hardly be stored in living organisms. Macromolecules and additives such as magnesium and phosphate are involved in the biogenic amorphous phases, acting as effective stabilizers [49]. Tao et al. suggested that the shell mineralization from the magnesium stabilized precursors is associated with the presence of Asp-rich proteins [50]. The Asp-rich proteins found in this study may switch on the calcium carbonate transformation from the amorphous to crystallized phases. Basic proteins provided a valuable balance to the classic view that acidic proteins control calcium carbonate deposition in nacre. Lys-rich matrix proteins (KRMPs) are the most basic proteins associated with the molluscan shell. The Lys-rich domain may interact with negatively charged ions (bicarbonate) or acidic matrix proteins. The Lys-rich matrix proteins are expressed only in the mantle edge, corresponding to the secretion of the prisms. The structure of KRMPs suggests that their function is to link the acidic soluble proteins to the hydrophobic framework of the prisms [6].

### Comparison of the transcriptomes between tissues secreting purple and white nacre

Digital expression profiling, also called tag sampling or RNA-seq, has proven to be a powerful and efficient approach for gene expression analysis at the genome level [51], and offers several advantages over microarray technologies [52]. Due to the rapid advances in next generation sequencing technologies, the digital expression profiling approach has become more widely used. Our digital expression profiling analysis identified a total of 358 differentially expressed genes, among which 137 showed significantly higher expression levels in the P-library and 221 showed significantly higher expression levels in the W-library (false discovery rate (FDR) <0.001). Among them, ten and twelve genes with functional annotations showed significantly higher expression levels in the P- and W-libraries, respectively. Intrinsic factor gene, which is crucial for the normal absorption of B_12_, was found to have higher expression levels in the P-library. Vitamin B_12_, also called cobalamin, is the only vitamin containing a metal element, and contains the biochemically rare element cobalt. It has been reported that more cobalt is contained in purple pearls than white pearls [46]. A Mg^2+^ and Co^2+^ transporter gene was also found in this study. Whether or not vitamin B_12_ metabolism is related to nacre color is an interesting question to pursue. In addition to the obvious difference in secreting nacre color between the two samples, purple nacre is considered to be more exquisite than white nacre, while white nacre is secreted faster than purple nacre. The secretion of the shell matrix was non synchronous in the temporal sequence between the two samples. A greater number of genes involved in modulating crystal polymorph were found to have higher expression levels in the P-library, while genes involved in basic crystal formation were found to have higher expression levels in the W-library. The differently expressed genes with function annotation mainly involved in signaling, cell proliferation, differentiation and apoptosis, cell-to-cell and cell-to-matrix interactions and extracellular matrix.

In vitro studies provided evidence for the presence of signal molecules in the nacre organic matrix, which are responsible for the recruitment of mammalian cells in the osteogenic pathway and bone cell activation, undergoing a complete sequence of mineralization. Retrieving like-proteins in the shell matrix of molluscs from distant taxa and bone nacre interactivity provides convergent data supporting the conservation of molecular signals for biomineralization control within the organic framework of biomineralized structures [53]. In this study, epidermal growth factor protein (EGFR) and bone morphogenetic protein1/Tolloid (BMP1/TLD)-like proteinase were found to have higher expression levels in the P-library. The EGFR signaling pathway is an important bone regulator and primarily plays an anabolic role in bone metabolism [54]. More recently, it has become apparent that the BMP1/TLD-like proteinases are activators of a broader subset of the TGF-β superfamily of proteins, with implications that these proteinases may be key in orchestrating the formation of ECM with growth factor activation and BMP signaling in morphogenetic processes [55]. Receptor tyrosine kinases (RTKs) are high-affinity cell surface receptors for many growth factors. One RTK, neurite outgrowth regulated kinase, was also found to have higher expression levels in the P-library. Semaphorins have been known as crucial regulators of morphogenesis and homeostasis over a wide range of organ systems. Cells of the periodontal attachment (cementoblasts, osteoblasts, and periodontal ligament fibroblasts) are descended from a common progenitor, the cranial neural crest. During their differentiation into different cell types, these cells separate from one another to form a laminated structure. Differential expression of semaphorins and plexins may be involved in regulating cell-sorting in the formation and regeneration of the periodontal attachment structure [56]. A common theme in the mechanism of semaphorin function is that they alter the cytoskeleton and the organization of actin filaments and the microtubule network. These effects occur primarily through the binding of semaphorins to their receptors, although transmembrane semaphorins also serve as receptors themselves. The best characterized receptors for mediating semaphorin signaling are members of the neuropilin and plexin families of transmembrane proteins [57]. In this study, semaphorin5A, semaphorin2a, semaphorin6, plexinA1, plexinA4 and plexinB were identified. Semaphorin5A was found to have especially high expression levels in the P-library. This result suggested that semaphorin signaling may play an important role in shell or pearl formation. Eukotriene B4 receptor and CD63 antigen were found to have higher expression levels in the P-library. Eukotriene B4 receptor increased the osteoclastic activity through the BLT1-Gi protein-Rac1 signaling pathway [58] and CD63 antigen mediated signal transduction events that play a role in the regulation of cell development, activation, growth and motility. Mitogen-activated protein kinase (MAPK) signaling cascades are organized hierarchically into three-tiered modules. MAPKs are phosphorylated and activated by MAPK-kinases (MAPKKs). In this study, one MAPKK was found to have higher expression levels in the W-library. MAPKs respond to extracellular stimuli and regulate gene expression [59]. Four transcription factors (Rho factor, DEAD box protein, pre-mRNA-splicing helicase BRR2 and RNA polymerase II transcription subunit) were also found to have higher expression levels in the W-library.

Genes modulating cell proliferation and differentiation, protein Tob1, Hillarin and cationic amino acid transporter protein were found to have higher expression levels in the P-library. The Tob1 gene encodes a member of the tob/btg1 family of anti-proliferative proteins that have a potential function in regulating cell growth. When exogenously expressed, Tob1 suppresses cell growth in tissue culture. Hillarin may function as a regulator of septin function during cytokinesis in the developing nervous system. Septin builds scaffolding to provide structural support during cell division and to compartmentalize parts of the cell. Human keratinocytes constitutively express cationic amino acid transporters 1 and 2, and cationic amino acid transporter mediated L-arginine influx, essential for inducible nitric oxide synthase and arginase enzyme activities, in turn modulates proliferation and differentiation of human epidermal skin cells [60].

The α-NAC (nascent polypeptide-associated complex) protein was specifically expressed in differentiated osteoblasts at the centers of ossification. Thus, the α-NAC gene product exhibits the properties of a developmentally regulated, bone-specific transcriptional coactivator [61]. NAC is a nascent dimeric complex composed of subunits alpha and beta. In the absence of its partner subunits, α-NAC negatively affects the growth of yeast [62]. In this study, one β-NAC protein was found to have higher expression levels in the P-library. IAP repeat-containing protein Mab21 was found to have higher expression levels in the W-library. Mab21 is an inhibitor of apoptosis that works by interfering with the activation of caspases.

Three genes that mediate cell-to-cell and cell-to-matrix interactions were found to have higher expression levels in the P-library. Thrombospondin 1, also known as THBS1, is a subunit of a disulfide-linked homotrimeric protein. This protein is an adhesive glycoprotein that mediates cell-to-cell and cell-to-matrix interactions. Ankyrins are a family of adaptor proteins that mediate the attachment of integral membrane proteins to the spectrin-actin based membrane skeleton [63]. This linkage is required to maintain the integrity of the plasma membranes and to anchor specific ion channels, ion exchangers and ion transporters in the plasma membrane. Integrins are receptors that mediate attachment between a cell and the tissues surrounding it, which may be other cells or the ECM. They also play a role in cell signaling and thereby regulate cellular shape, motility, and the cell cycle.

Pearl and mollusk shell are the biomineralization products of CaCO_3_ crystals, matrix proteins and other biopolymers. Initially, insoluble matrix proteins form a framework structure and provide a nucleation sheet for the first nucleation of highly oriented crystals. Then, calcium ions are assembled by water-soluble matrix proteins and deposit in orderly arrays on the nucleation sites of the framework [64]. Actin forms microfilaments which are typically one of the most dynamic of the three subclasses of the eukaryotic cytoskeleton. Filamins are a class of proteins that hold two actin filaments at large angles. A coiled coil is a structural motif in proteins, in which 2–7 alpha-helices are coiled together like the strands of a rope. Coiled coils are abundant structures found in a diverse array of proteins, from transcription factors such as Jun and Fos, involved in cell growth and proliferation, to Matrilins, involved in the development of cartilage and bone [65]. Matrilins may play a role in stabilizing the extracellular matrix structure, because they can self-associate into supramolecular structures, resulting in the formation of filamentous networks [66]. In this study, one actin gene, one Filamin-like protein gene and one coiled-coil domain containing 59 genes (CCDC59) were found to have higher expression levels in the W-library. Reelin is a large secreted extracellular matrix protein. Reelin expression has recently been associated with abnormal bone remodeling of the otic capsule in the pathogenesis of otosclerosis. Hypermethylation of the reelin promoter is often seen during aging and is associated with its reduced expression [67]. Dermatopontin, also known as TRAMP, is a widely expressed noncollagenous protein component of the extracellular matrix. It regulates the interaction of TGF-beta and decorin and is involved in collagen matrix organization. In mollusks, Dermatopontin is considered a major component of the shell matrix proteins [43]. In this study, the reelin and dermatopontin genes were found to have higher expression levels in the W-library. Calponin tonically inhibits the ATPase activity of myosin in smooth muscle. Calponin is also thought to negatively affect the bone making process because it is expressed in high amounts in osteoblasts [68]. In this study, the calponin gene was found to have higher expression levels in the P-library. One BNR containing protein and one Whey acidic protein were also found to have higher expression levels in the P-library. It was suggested that Asp residues in the organic matrices interacted with calcium atoms in calcium carbonate in order to regulate the crystal polymorph [69], [70].

Nacrein is the most studied molluscan shell protein family in many aspects. In situ hybridization studies show that nacrein is ubiquitous and probably displays the same functions in prismatic and nacreous layers [6]. In this study, one gene similar to *Pinctada margaritifera* nacrein B3 (HQ654771.1) and one gene similar to *Pinctada margaritifera* nacrein B4 (AEC03972.1) were found to have higher expression levels in the P- and W-libraries, respectively. This difference in expression implies that nacrein B3 and B4 are different in function. Nacrein related proteins contain two functional domains, a carbonic anhydrase-like domain (CA domain) and a domain with a repeated sequence rich in Asn and Gly (NG-repeat domain). CA is thought to be one of the most important enzymes responsible for shell formation by catalyzing the conversion of CO_2_ to HCO_3_^−^, thus creating a favorable environment for crystal nucleation. The deposition of calcium carbonate is accompanied by the release of protons, which have to be reabsorbed by the calcifying epithelium for precluding acidification of the extrapallial fluid and possible resolubilization of the newly formed minerals. It has been suggested that proton pumps are involved in extruding protons from the extrapallial fluid toward the cytosol. However, this process is poorly documented for mollusks. In this study, one V-ATPase gene was found to have higher expression levels in the W-library. It has also been suggested that proton pumps work in the reverse direction in some cases, inducing acidosis in the extrapallial space [6].

### The identification of Simple Sequence Repeats (SSRs) and Single Nucleotide Polymorphisms (SNPs)

Both SSRs and SNPs are valuable markers for aquaculture breeding programs. It has been reported that approximately 3–7% of expressed genes contain putative SSR motifs, mainly within the untranslated regions of the mRNA [17]. SSR markers derived from EST sequences have been extensively used in constructing genetic maps of molluscan species [21]. In this study, we performed a general screen on the freshwater pearl mussel unisequences dataset for the presence of SSRs. A total of 9,474 SSRs were found in 8,100 unisequences. The major types of the identified SSRs were dinucleotide (4,443) and trinucleotide (3,034), followed by tetranucleotide (1,770), hexanucleotide (180) and pentanucleotide (47). The most frequent SSR motif was AC/GT (1,826), followed by AT/TA (1,705), AAT/ATT (1,497), ACAT/ATGT (1,184), AG/CT (910) and AAC/GTT (730). The complete list of SSR information is provided in File S1.

Because the ESTs generated in the present study using the 454 technology are from two different samples, we expected SNPs to be present in our EST collection. We identified a total of 27,303 SNPs between the P- and W-libraries, among which 13087 were transitions, 9,944 were transversions, and 4,272 were indels (Figure S2). In summary, the SSRs and SNPs identified in this study provided a valuable resource for future studies on the analysis of interesting traits and genetic linkage mapping in the freshwater pearl mussel.

## Conclusions

The comparative transcriptome analysis identified 358 differentially expressed genes that are potentially involved in nacre coloring. This study provided invaluable new data to assist in our understanding of the mechanisms of nacre coloring, pearl or shell formation and shell evolution. The molecular markers identified in this study will provide a material basis for future genetic linkage and quantitative trait loci analyses, and will be essential for accelerating aquaculture breeding programs with this species.

## Supporting Information

File S1 **The list of SSRs information indentified from all the unisequences.**
(XLSX)

Figure S1 **KEGG analysis of deduced protein sequences.**
(TIF)

Figure S2 **The number of different type SNPs identified from all the EST.**
(TIF)
