# Supplementary figures and images for: Comparative Analysis of the Transcriptome in Tissues Secreting Purple and White Nacre in the Pearl Mussel Hyriopsis cumingii
Source: PLoS One. 2013 Jan 14;8(1):e53617. doi: 10.1371/journal.pone.0053617 (PMC3544910; doi:10.1371/journal.pone.0053617)

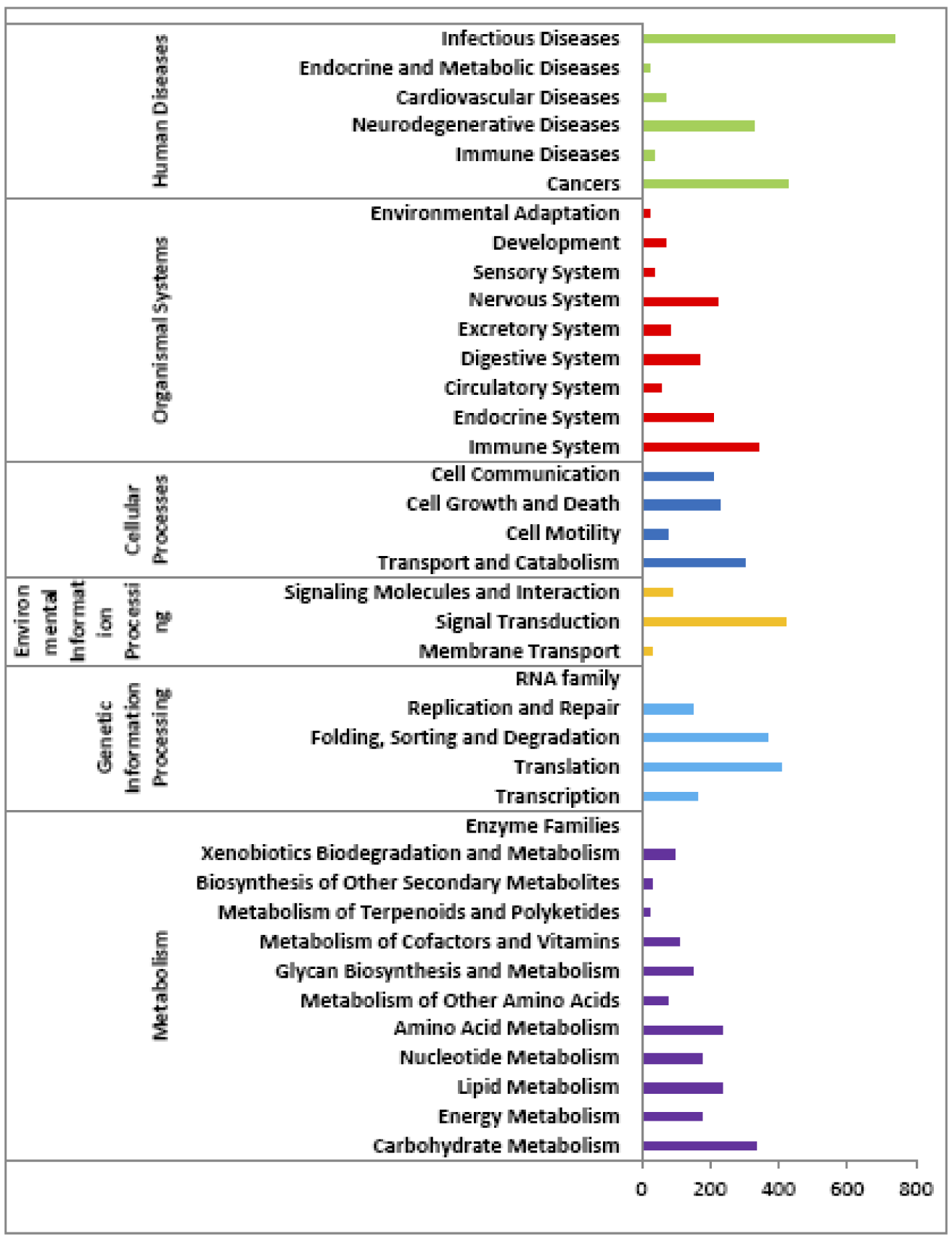

Supplement: Figure S1 — KEGG analysis of deduced protein sequences. (TIF) [file pone.0053617.s002.tif]

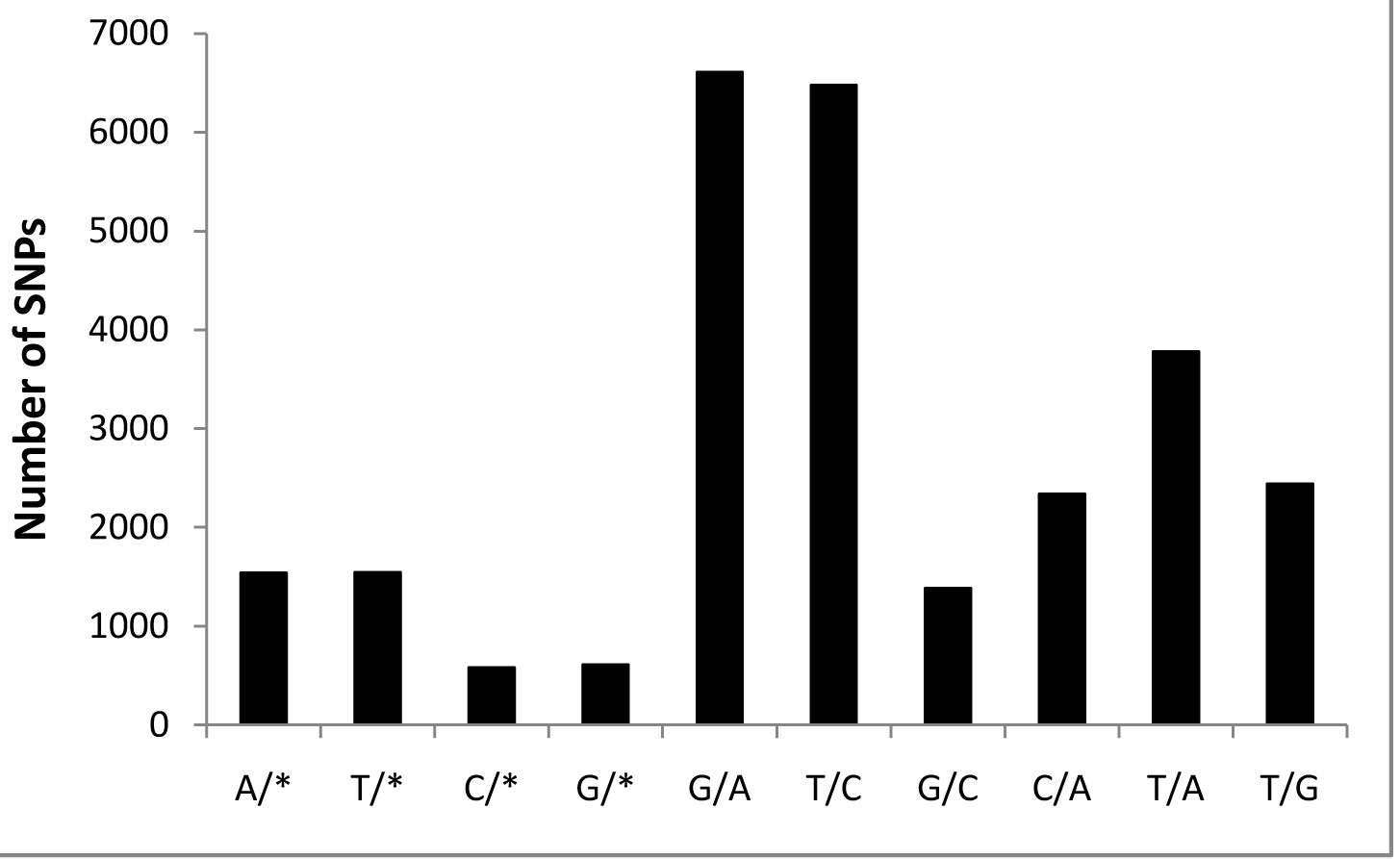

Supplement: Figure S2 — The number of different type SNPs identified from all the EST. (TIF) [file pone.0053617.s003.tif]
